# Supplementary material for: Topologically ordered time crystals
Source: Nat Commun. 2024 Nov 13;15:9845. doi: 10.1038/s41467-024-54086-4 (PMC11561053; doi:10.1038/s41467-024-54086-4)
Supplement: Supplementary file 1 — Supplementary Information [file 41467_2024_54086_MOESM1_ESM.pdf]

Supplementary Information for "Topologically ordered time crystals"

Thorsten B. Wahl,[1] Bo Han,[2,3] and Benjamin Béri[1,3,*]

[1] DAMTP, University of Cambridge, Wilberforce Road, Cambridge, CB3 0WA, UK
[2] Department of Condensed Matter Physics, Weizmann Institute of Science, Rehovot 7610001, Israel
[3] T.C.M. Group, Cavendish Laboratory, University of Cambridge, J.J. Thomson Avenue, Cambridge, CB3 0HE, UK

In the Supplementary Information, we provide further details on the Floquet spectrum and signatures of topologically ordered time crystals (TTCs), and illustrate these using numerical simulations of surface-code TTCs. Our numerical findings provide further support to the main text's analytical arguments for the robustness of TTCs, and suggest the feasibility of detecting TTCs in programmable quantum devices.

Supplementary Note 1: Floquet spectrum and the robustness of TTCs

For a static Hamiltonian $H = \sum_P J_P S_P + \delta H$, where S_P are check operators for a nonchiral Abelian topological order (TO) and J_P are disordered, a (pre-thermal) many-body localized (MBL) phase is expected for any δH that features only local terms with couplings of order $g \ll \delta J$ (where δJ is the width of the J_P distribution) [1–4].

We now review, building on TC considerations [5–7], how such a H arises as an effective Floquet Hamiltonian for the evolution over two periods, $U_{\mathrm{F}}^2 \approx \exp[-i(\sum_P 2J_P S_P + \delta H)]$. We consider

$$U_{\mathrm{F}} = \overline{O} \exp(-i\delta H_1) \exp(-iH_0), \tag{1}$$

where $H_0 = \sum_P J_P S_P$, and δH_1 features only local terms with couplings of order $g \ll \delta J$. To simplify our analytical considerations, instead of using $U_{\mathrm{F}} = \exp(-iH_1)\exp(-iH_0)$ with H_1 including a term $\frac{\pi}{2}\sum_{j\in\gamma_X} X_j$, here we defined U_{F} by explicitly separating the factor $\overline{O} = \prod_{j\in\gamma_X} X_j$. This is equivalent to $U_{\mathrm{F}} = \exp(-iH_1)\exp(-iH_0)$ when δH_1 and $\frac{\pi}{2}\sum_{j\in\gamma_X} X_j$ commute, and the difference is inconsequential for our conclusions otherwise. [To further support this, our numerical simulations will feature drives of the form $U_{\mathrm{F}} = \exp(-iH_1)\exp(-iH_0)$.]

Using that Pauli strings either commute or anticommute, we split $\delta H_1 = \delta H_1' + \delta H_1''$ into a term $\delta H_1'$ that commutes with $\overline{O}$ and a term $\delta H_1''$ that anticommutes with $\overline{O}$. Using $\overline{O}^2 = \mathbb{1}$ and $\overline{O} H_0 = H_0 \overline{O}$, we have

$$U_{\mathrm{F}}^2 = e^{-i(\delta H_1' - \delta H_1'')} e^{-iH_0} e^{-i(\delta H_1' + \delta H_1'')} e^{-iH_0} = e^{-i(2H_0 + \widehat{\delta H})}, \tag{2}$$

where a formal expression for $\widehat{\delta H}$ can be obtained using the Baker-Campbell-Hausdorff (BCH) formula. Assuming $g, |J_P| \ll 1$ (and still $g \ll \delta J$) and denoting by δH the truncation of the BCH expansion for $\widehat{\delta H}$ at low orders leads to $U_{\mathrm{F}}^2 \approx \exp[-i(\sum_P 2J_P S_P + \delta H)]$, with δH featuring only local couplings with coefficients of order $g \ll \delta J$.

This suggests that U_{F} is TO MBL, which, by Proposition 1 of the main text, then implies the robustness of TTCs. It is however only a qualitative argument, based on a truncated BCH expansion. Hence, we shall test the robustness of TTCs through the Floquet spectrum. Before turning to numerical simulations (see Supplementary Note 1 B), we first provide further details on the theory, and compare to the static case. Following Proposition 1 of the main text, but allowing for finite $L_\perp$ (the length of the shortest γ_X, henceforth understood as the number of qubits in γ_X, i.e., the weight $|\gamma_X|$ of $\overline{O} = \prod_{j\in\gamma_X} X_j$), we have

$$U_{\mathrm{F}} = \widetilde{\mathcal{O}} e^{-if(\{T_P, \widetilde{\mathcal{O}}\})}, \quad f(\{T_P, \widetilde{\mathcal{O}}\}) = f_0(\{T_P\}) + \widetilde{\mathcal{O}}\, \Delta_\pi\, f_1(\{T_P\})/2, \tag{3}$$

where f_0 is exponentially local, $\widetilde{\mathcal{O}}\, f_1$ is exponentially local in terms of the operators $\widetilde{\mathcal{O}}\{T_P\}$, and $\Delta_\pi \sim \exp(-L_\perp/\xi)$, where we absorb numerical prefactors in the exponential into the localization length ξ. The eigenstates $|\psi_{\mathbf{s},o}\rangle$ of U_{F} are simultaneous eigenstates of the T_P and $\widetilde{\mathcal{O}}$, with corresponding eigenvalues in the vector $\mathbf{s}$ and o, with $s_i, o \in \{-1, 1\}$. Hence the Floquet spectrum is

$$e^{-i\varepsilon_{\mathbf{s},o}} = o\, e^{-if(\mathbf{s},o)} = o\, e^{-if_0(\mathbf{s})} e^{-io\,\Delta_\pi\, f_1(\mathbf{s})/2}. \tag{4}$$

For $L_\perp/\xi \to \infty$ we have $\Delta_\pi \to 0$, hence the Floquet spectrum consists of opposite pairs $\pm e^{-if_0(\mathbf{s})}$. In terms of the relative phase between the levels in such a pair, the Floquet spectrum is π-paired. For finite $L_\perp/\xi$, this relative phase gets a correction $\Delta_\pi\, f_1(\mathbf{s})$ but this correction decays exponentially in $L_\perp/\xi$.

A. Comparison to static TO MBL

As noted in the main text, f in Supplementary Eq. (3) is agnostic to the length $L_\parallel$ of the shortest γ for the logical $\mathcal{Z}_\gamma = \prod_{j\in\gamma} Z_j$ conjugate to $\overline{O}$. This signifies the TTC form of absolute stability. We now give further details on the comparison of this feature to static TO MBL. In the latter case, $U_{\rm F} = e^{-iH}$, where H can be written as [1–4]

$$H = h_0(\{T_P\}) + c_1\widetilde{\mathcal{O}}\, h_1(\{T_P\}) + c_2\widetilde{\mathcal{W}}_\gamma\, h_2(\{T_P\}) + c_1(i\widetilde{\mathcal{O}}\widetilde{\mathcal{W}}_\gamma)\, h_3(\{T_P\}), \tag{5}$$

where h_α are exponentially local in the sense analogous to f_1 in Supplementary Eq. (3), c_1 decays exponentially with $L_\perp/\xi$, c_2 with $L_\parallel/\xi$, and c_3 with both $L_\perp/\xi, L_\parallel/\xi$. The key difference between Supplementary Eq. (3) and Supplementary Eq. (5) is that in the former only $\widetilde{\mathcal{O}}$ enters, but not $\widetilde{\mathcal{W}}_\gamma$ because already in the unperturbed limit $U_{\rm F0}$ features $\overline{O}$ and this specifies the unperturbed eigenbasis in logical space. Under MBL, perturbations dress this but the Floquet unitary continues to have the form in Supplementary Eq. (3), with $\widetilde{\mathcal{O}}$ specifying the logical eigenbasis.

By contrast, in the static case, the unperturbed Hamiltonian features no logical operators, hence its logical eigenbasis is arbitrary. Hence the unperturbed spectrum is degenerate and this degeneracy is split by perturbations such that the eigenbasis in the logical subspace is generally different for different $\mathbf{s}$. This $\mathbf{s}$ dependent perturbed logical eigenbasis is encoded by the appearance of $\widetilde{\mathcal{O}}$, $\widetilde{\mathcal{W}}_\gamma$, and $i\widetilde{\mathcal{O}}\widetilde{\mathcal{W}}_\gamma$ with generally distinct $\{T_P\}$ dependence of their respective h_α.

The spectrum of H is degenerate for $L_\perp/\xi, L_\parallel/\xi \to \infty$; the degeneracies are split (by order c_α) if either or both of $L_\perp/\xi, L_\parallel/\xi$ is finite. This is distinct from Supplementary Eq. (3), where the π-pairing in the spectrum receives corrections only from finite $L_\perp/\xi$, but not from finite $L_\parallel/\xi$.

B. Numerical results

We next study the Floquet spectrum numerically. We consider geometries illustrated in Supplementary Fig. 1, with $N = N_x \times N_z$ qubits with N_z odd, where $N_x = L_\perp$ and $N_z = L_\parallel$. The Floquet unitary we use is

$$U_{\rm F} = e^{-iH_1}e^{-iH_0}, \quad H_0 = \sum_P J_P S_P, \quad H_1 = \sum_j \left(\frac{\pi}{2} + g_j^{(X)}\right) X_j + g_j^{(Y)} Y_j + g_j^{(Z)} Z_j, \tag{6}$$

where S_P are the surface-code check operators and X_j, Y_j, Z_j are the Pauli X, Y, Z operators on qubit j, respectively. We sample J_P uniformly from the interval $]-\pi,\pi]$, independently for each P. We sample the direction of $(g_j^{(X)}, g_j^{(X)}, g_j^{(X)})$ uniformly on the unit sphere and its length uniformly from $[0,g]$, independently for each j.

For $g=0$, the Floquet unitary $U_{\rm F}$ in Supplementary Eq. (6) realizes an unperturbed TTC, since the systems we consider (by having either X-checks or Z-checks, Z-checks featuring an even number of qubits, and N_z being odd) satisfy the sufficient conditions noted in the main text for $\exp(-i\frac{\pi}{2}\sum_j X_j)$ to realize a logical $\overline{X}$. By the robustness of MBL, the TTC is expected to persist for $0 < g \ll 1$. In such a perturbed TTC, with $L_\perp$ finite, the π spectral pairing receives corrections of order $\Delta_\pi \sim \exp(-L_\perp/\xi)$.

While accessing $L_\perp/\xi$ directly is difficult, the exponential decay of Δ_π can be understood perturbatively: $\Delta_\pi \sim g^{L_\perp}$ for small g because it takes (at least) order $L_\perp$ in perturbation theory for the X_j perturbations to combine into $\overline{O}$. To test this scaling numerically, we define [8] $\Delta_0^i = \varepsilon_{i+1} - \varepsilon_i$, with ε_i the $i^{\rm th}$ Floquet energy and $\Delta_\pi^i = |\varepsilon_{i+D_{\rm H}/2} - \varepsilon_i - \pi|$, where $D_{\rm H} = 2^N$ is the Hilbert space dimension for N qubits. The level $\varepsilon_{i+D_{\rm H}/2}$ is thus halfway across the spectrum from ε_i. The typical values $\langle \ldots \rangle_{\rm typ} = \exp(\langle \log(\ldots)\rangle)$ are expected to follow $\langle \Delta_0\rangle_{\rm typ} \sim 1/D_{\rm H}$ and $\langle \Delta_\pi\rangle_{\rm typ} \sim g^{L_\perp}$. In

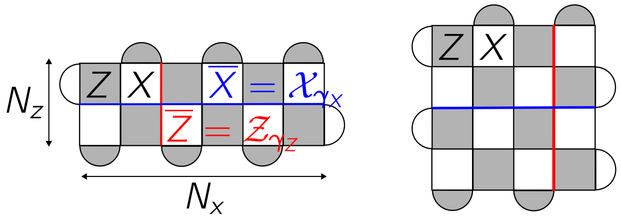

Supplementary Figure 1. Geometry for the surface code TTCs considered in the numerical simulations. On the left is an example with $N_z = 3$, $N_x = 7$. The example on the right has $N_z = N_x = 5$. $N = N_x N_z = 25$ is our largest numerically accessible system size for state evolution, studied in Supplementary Notes 2,3. The largest system size for computing the Floquet spectrum is $N = 12$.

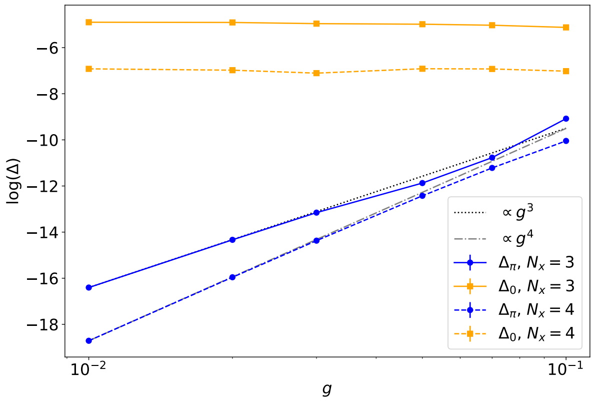

Supplementary Figure 2. The log-average many-body spacing Δ_0 and deviation Δ_π from π-pairing for the TTC Floquet unitary in Supplementary Eq. (6). We consider $N_z = L_\parallel = 3$ and $N_x = L_\perp = 3, 4$. The angular brackets $\langle \ldots \rangle$ denote averaging over all eigenstates and 100 disorder realizations. We also show $g^{L_\perp}$ as a guide for the eye. Error bars (imperceptible) show the standard error of the log-average.

our numerics, we take g sufficiently small for $\Delta_\pi < \Delta_0$ and thus for π spectral pairs to be identifiable in the many-body spectrum. We show our numerical results in Supplementary Fig. 2. The results are consistent with $\langle \Delta_\pi \rangle_{\text{typ}} \sim g^{L_\perp}$, even for $L_\perp > L_\parallel$, illustrating that π pairing receives corrections from finite $L_\perp$ but not from finite $L_\parallel$.

These results already suggest the robustness of TTCs. In Supplementary Note 2 A we further explore this robustness, studying larger systems accessible via state evolution.

Supplementary Note 2: Eigenstate-averaged autocorrelators via quantum typicality

Quantum typicality [9, 10] is a powerful approach to detect eigenstate-averaged expectations. It was, in particular, used to experimentally detect time-crystalline autocorrelators in ordinary TCs using the Google Sycamore processor [7]. As we discuss below, it is similarly suitable to detect the autocorrelator

$$C(m;\gamma) = \frac{1}{D_{\mathrm{H}}} \sum_{\alpha} C_\alpha(m;\gamma) = \frac{1}{D_{\mathrm{H}}} \mathrm{Tr}\left[\mathcal{W}_\gamma(mT)\mathcal{W}_\gamma(0)\right], \tag{7}$$

where $C_\alpha(m;\gamma) = \langle \alpha | \mathcal{W}_\gamma(mT)\mathcal{W}_\gamma(0) | \alpha \rangle$ with Floquet eigenstate $|\alpha\rangle$ and D_{H} is the dimension of the Hilbert space.

Quantum typicality is the finding that, for any observable A, and for states $|\phi\rangle$ sampled from the uniform distribution of normalized states within the Hilbert space (i.e., from the Haar distribution), we have [9, 10]

$$\mathbb{E}_\phi\left(\langle \phi | A | \phi \rangle\right) = \langle A \rangle_\infty, \quad \mathrm{Var}_\phi\left(\langle \phi | A | \phi \rangle\right) = \frac{1}{D_{\mathrm{H}} - 1}\left(\langle A^2 \rangle_\infty - \langle A \rangle_\infty^2\right), \tag{8}$$

where $\mathbb{E}_\phi$ and Var_ϕ are the Haar average and variance, respectively, and $\langle A \rangle_\infty = \frac{1}{D_{\mathrm{H}}}\mathrm{Tr}A$. With $A = \mathcal{W}_\gamma(mT)\mathcal{W}_\gamma(0)$, in particular, $\langle A \rangle_\infty$ is the desired eigenstate-averaged autocorrelator.

For a system of N qubits we have $D_{\mathrm{H}} = 2^N$, hence the variation of $\langle \phi | A | \phi \rangle$ with $|\phi\rangle$ is suppressed as $2^{-N/2}$ for $N \gg 1$. This allows one to probe $\langle A \rangle_\infty$ to high accuracy using just a few (or even a single) $|\phi\rangle$. A Haar random $|\phi\rangle$ is a generic highly-entangled state and such states can be prepared in experiments, e.g., as $|\phi\rangle = U_{\mathrm{E}} |b\rangle$ using a suitable entangling circuit U_{E} acting on an initial computational basis (hence product) state $|b\rangle$ defined by a bitstring b [7]. The choice $A = \mathcal{W}_\gamma(mT)\mathcal{W}_\gamma(0)$ can be implemented by adapting the interferometric approach of Ref. 7 used for ordinary TCs. There, $A = Z_j(mT)Z_j(0)$ was implemented using a controlled Z_j with a measurement ancilla as control [7]; for TTCs the multi-qubit operator $A = \mathcal{W}_\gamma(mT)\mathcal{W}_\gamma(0)$ requires a controlled $\mathcal{W}_\gamma$ instead. For $\mathcal{W}_\gamma = \mathcal{Z}_\gamma = \prod_{j\in\gamma} Z_j$ this is a $|\gamma|$-qubit controlled Z gate. Such gates, up to at least $|\gamma| = 6$, should be feasible, since controlled 6-qubit Pauli operators have already been realized, e.g., in the demonstration of surface-code anyon braiding in the Google Sycamore [11].

A. Numerical results

We next show numerical results on

$$C_\phi(m;\gamma) = \langle\phi|\, \mathcal{Z}_\gamma(mT)\mathcal{Z}_\gamma(0)\, |\phi\rangle \tag{9}$$

for a surface-code TTC using the Floquet unitary in Supplementary Eq. (6). We evaluate $C_\phi(m;\gamma)$ using state evolution; this allows us to access larger system sizes than we could for the Floquet spectrum. The correction $\Delta_\pi \sim \exp(-L_\perp/\xi)$ to π-pairing translates to a finite, but exponentially long, lifetime $\sim \Delta_\pi^{-1}$ of the time-crystalline signal in $C(m;\gamma)$: the small, and random, deviations from π phase difference translate to random phases of typical magnitude $m\Delta_\pi$, and these cause the time-crystal signal to dephase, and hence decay, once $m\Delta_\pi \sim 1$.

In Supplementary Fig. 3, left panel, we focus on $N_x = N_z = 5$ (with $N = 25$ these are the largest systems we could numerically access) and study the range of g consistent with TTC physics. The results suggest that, for $g \lesssim 0.3$ and after an initial decay, the magnitude of the disorder-averaged $C_\phi(m;\gamma)$ remains roughly constant in the $m \leqslant 200$ time window, consistent with the $\Delta_\pi^{-1} \sim \exp(L_\perp/\xi)$ TTC lifetime. For $g > 0.3$, by contrast, the data suggest an exponentially decaying $C_\phi(m;\gamma)$, indicative of the breakdown of the TTC phase. The $N_x = N_z = 5$ data thus suggest that the TTC phase is robust, and persists for $g \lesssim 0.3$. (The requirement $g/\delta J = g/\pi \lesssim 0.1$ for a TTC is similar to the conditions found numerically for static TO MBL [12].) We next numerically test some features of the TTC, focusing on $g = 0.2$.

In Supplementary Fig. 3, middle panel, we study $C_\phi(m;\gamma)$ for $N_z = |\gamma| = 3$, and $N_x = L_\perp = 3, 4, 5, 7$. The data are consistent with the exponential increase of the lifetime with $L_\perp$. The numerical observation of this characteristic TTC feature also provides further evidence of the robustness of the TTC phase against perturbations.

We next turn to probing the perimeter law [1, 2, 13, 14], in its TTC incarnation, via $C_\phi(m;\gamma)$. For this, we focus on $N_z = 3$, $N_x = L_\perp = 8$ and consider $|\gamma| = 3, 5, 7$. The results, shown in Supplementary Fig. 3, right panel, are consistent with the exponential decrease of the time crystalline signal with $|\gamma|$. As our results illustrate, for sufficiently small g, the signal remains of appreciable magnitude for a range of $|\gamma|$. This provides support for the TTC perimeter law being detectable in experiments.

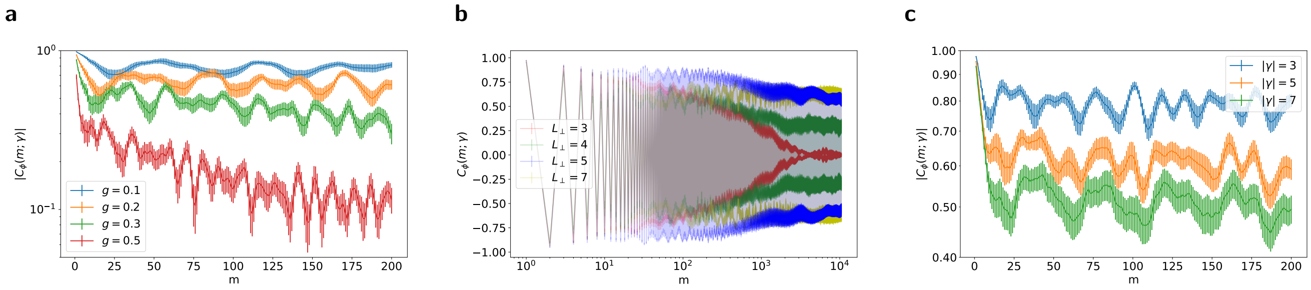

Supplementary Figure 3. Numerical results on the autocorrelator $C_\phi(m;\gamma)$. In all panels, we show averages over 10 disorder realizations and 5 states ϕ per realization, and the error bars show the standard error of the mean. Left panel: Results for a square geometry, for the largest numerically accessible system $N_x = N_z = 5$, for $|\gamma| = 5$ and $g = 0.1, 0.2, 0.3, 0.5$. Middle panel: increasing the lifetime of the TTC signal by increasing $L_\perp$. The results are for $N_z = |\gamma| = 3$, $g = 0.2$, and $N_x = L_\perp = 3, 4, 5, 7$. Right panel: perimeter law, tested for $g = 0.2$, $|\gamma| = 3, 5, 7$ and $N_z = 3$, $N_x = L_\perp = 8$.

Supplementary Note 3: Spatiotemporal correlators via approximate eigenstates

We next discuss approximating

$$C_\alpha(m;\gamma,\gamma') = \langle\alpha|\, \mathcal{W}_\gamma(mT)\mathcal{W}_{\gamma'}\, |\alpha\rangle = \sum_\beta e^{i(\varepsilon_\alpha - \varepsilon_\beta)m} \langle\alpha|\, \mathcal{W}_\gamma\, |\beta\rangle \langle\beta|\, \mathcal{W}_{\gamma'}\, |\alpha\rangle \,, \tag{10}$$

in the strongly MBL phase where the unperturbed TTC eigenstate $|\mathbf{s}, o\rangle$ well approximates a Floquet eigenstate $|\alpha_{\mathbf{s},o}\rangle$. We therefore consider

$$C_{s,o}(m,n;\gamma,\gamma') = \langle\mathbf{s}, o|\, \mathcal{W}_\gamma[(m+n)T]\, \mathcal{W}_{\gamma'}(nT)\, |\mathbf{s}, o\rangle \,. \tag{11}$$

In the expansion $|\mathbf{s}, o\rangle = \sum_\alpha b_\alpha |\alpha\rangle$, Floquet eigenstates $|\alpha\rangle \neq |\alpha_{\mathbf{s},o}\rangle$ contribute perturbatively, with coefficients decreasing in magnitude with increasing the number of LIOMs with eigenvalues flipped from their value in $|\alpha_{\mathbf{s},o}\rangle$ [12].

Hence, for $n \gg 1$, off-diagonal ($\alpha' \neq \alpha$) terms in

$$C_{s,o}(m,n;\gamma,\gamma') = \sum_{\alpha,\alpha'} b^*_{\alpha'} b_\alpha \langle\alpha'| \mathcal{W}_\gamma[(m+n)T]\, \mathcal{W}_{\gamma'}(nT)\, |\alpha\rangle\,, \tag{12}$$

suffer both dephasing suppression [15] and perturbative suppression.

Focusing on $d(\gamma,\gamma') \gg \xi$, we recall the expansion $\mathcal{W}_{\gamma^{(\prime)}} = \mathcal{S}_{0\gamma^{(\prime)}} + \mathcal{S}_{1\gamma^{(\prime)}} + \mathcal{S}_{x\gamma^{(\prime)}}$, where $\mathcal{S}_{0\gamma^{(\prime)}}$ is a linear combination of various $\prod_P T_P$, $\mathcal{S}_{1\gamma^{(\prime)}}$ is that of various $\widetilde{\mathcal{W}}_{\gamma^{(\prime)}} \prod_P T_P$, and $\mathcal{S}_{x\gamma^{(\prime)}}$ is similar but with the product featuring at least one T^x_P. By the transversal quasilocality of $\mathcal{W}_{\gamma^{(\prime)}}$, a T^x_P in the expansion of $\mathcal{W}_{\gamma'}$ cannot be undone by one in $\mathcal{W}_\gamma$ and vice versa. Hence, the T^x_P enter only via offdiagonal terms in Supplementary Eq. (12), thus, analogously to their fate for Supplementary Eq. (10) (cf. main text, Methods) but now via perturbative and dephasing mechanisms, the $\mathcal{S}_{x\gamma}$ contributions are suppressed relative to $\mathcal{S}_{0\gamma^{(\prime)}}$ and $\mathcal{S}_{1\gamma^{(\prime)}}$. Using Supplementary Eq. (12), with $n \gg 1$, one thus approximately obtains

$$C_{s,o}(m,n;\gamma,\gamma') \approx \sum_\alpha |b_\alpha|^2 \langle\alpha| \mathcal{W}_\gamma[(m+n)T]\, \mathcal{W}_{\gamma'}(nT)\, |\alpha\rangle \approx |b_{\alpha_{s,o}}|^2 \langle\alpha_{\mathbf{s},o}| \mathcal{W}_\gamma[(m+n)T]\, \mathcal{W}_{\gamma'}(nT)\, |\alpha_{\mathbf{s},o}\rangle\,. \tag{13}$$

This allows one to study the TTC perimeter law, including the absence of a decay with $d(\gamma,\gamma')$.

We next study the TTC perimeter law using $C_{s,o}(m,n;\gamma,\gamma')$ for $n \gg 1$. To study features arising from a generic choice $|\mathbf{s},o\rangle$, one can consider the average over a set of randomly chosen $|\mathbf{s},o\rangle$. In doing this, care must however be taken to eliminate random signs that arise because $\widetilde{\mathcal{W}}_\gamma \widetilde{\mathcal{W}}_{\gamma'} = \prod_{P\in A_{\gamma\gamma'}} T_P$, with $A_{\gamma\gamma'}$ the area enclosed by γ and γ': as $|\mathbf{s},o\rangle$ sets the T_P eigenvalues (via $\mathbf{s}$), to a good approximation, random choices of $|\mathbf{s},o\rangle$ result in random signs in $C_{s,o}(m,n;\gamma,\gamma')$. These signs can be eliminated using that the T_P, and hence these signs, are time independent. This suggests to study the average of

$$\widehat{C}_{s,o}(m,n;\gamma,\gamma') = \frac{C_{s,o}(m,n;\gamma,\gamma')}{C_{s,o}(0,n;\gamma,\gamma')} |C_{s,o}(0,n;\gamma,\gamma')| = C_{s,o}(m,n;\gamma,\gamma')\, \mathrm{sgn}[C_{s,o}(0,n;\gamma,\gamma')], \tag{14}$$

instead of the average of $C_{s,o}(m,n;\gamma,\gamma')$.

A. Numerical results

In Supplementary Fig. 4, we show $\widehat{C}_{s,o}(m,n;\gamma,\gamma')$, with $n = 100$, obtained by state evolution using Supplementary Eq. (6) with $g = 0.2$, $N_z = 3$, and $N_x = L_\perp = 8$, and averaging over 5 randomly chosen $|\mathbf{s},o\rangle$ and 10 disorder realizations. The data show a clear period-doubled component, and exponential decay with the perimeter $|\gamma| + |\gamma'|$, and no considerable decay with the separation $d(\gamma,\gamma')$. Hence the results are consistent with the TTC perimeter law.

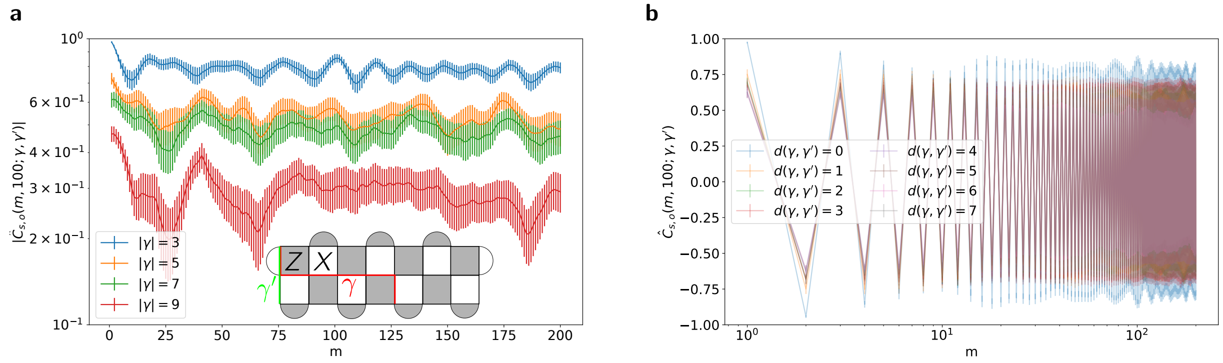

Supplementary Figure 4. Numerical results on $\widehat{C}_{s,o}(m,100;\gamma,\gamma')$. We consider $g = 0.2$, $N_z = 3$, and $N_x = L_\perp = 8$, and average over 5 randomly chosen $|\mathbf{s},o\rangle$ and 10 disorder realizations. In all panels, the error bars show the standard error of the mean. Left panel: data with $|\gamma'| = 3$, $|\gamma| = 3,5,7,9$. The paths γ and γ' are shown in the inset; $|\gamma|$ is set by the length of the horizontal path segment. Right panel: data with $|\gamma'| = |\gamma| = 3$, with $d(\gamma,\gamma') = 0,1,\ldots,7$. The results are consistent with the TTC perimeter law.

* bfb26@cam.ac.uk

[1] D. A. Huse, R. Nandkishore, V. Oganesyan, A. Pal, and S. L. Sondhi, "Localization-protected quantum order," Phys. Rev. B **88**, 014206 (2013).
[2] B. Bauer and C. Nayak, "Area laws in a many-body localized state and its implications for topological order," J. Stat. Mech. **2013**, P09005 (2013).
[3] A. C. Potter and A. Vishwanath, "Protection of topological order by symmetry and many-body localization," arXiv:1506.00592.
[4] T. B. Wahl and B. Béri, "Local integrals of motion for topologically ordered many-body localized systems," Phys. Rev. Res. **2**, 033099 (2020).
[5] N. Y. Yao, A. C. Potter, I.-D. Potirniche, and A. Vishwanath, "Discrete time crystals: Rigidity, criticality, and realizations," Phys. Rev. Lett. **118**, 030401 (2017).
[6] D. V. Else, B. Bauer, and C. Nayak, "Prethermal phases of matter protected by time-translation symmetry," Phys. Rev. X **7**, 011026 (2017).
[7] X. Mi *et al.*, "Time-crystalline eigenstate order on a quantum processor," Nature **601**, 531–536 (2022).
[8] C. W. von Keyserlingk, V. Khemani, and S. L. Sondhi, "Absolute stability and spatiotemporal long-range order in Floquet systems," Phys. Rev. B **94**, 085112 (2016).
[9] S. Popescu, A. J. Short, and A. Winter, "Entanglement and the foundations of statistical mechanics," Nat. Phys. **2**, 754–758 (2006).
[10] S. Goldstein, J. L. Lebowitz, R. Tumulka, and N. Zanghì, "Canonical typicality," Phys. Rev. Lett. **96**, 050403 (2006).
[11] K. J. Satzinger, Y. Liu, A. Smith, C. Knapp, M. Newman, C. Jones, *et al.*, "Realizing topologically ordered states on a quantum processor," Science **374**, 1237–1241 (2021).
[12] F. Venn, T. B. Wahl, and B. Béri, "Many-body-localization protection of eigenstate topological order in two dimensions," arXiv:2212.09775.
[13] K. G. Wilson, "Confinement of quarks," Phys. Rev. D **10**, 2445 (1974).
[14] J. B. Kogut, "An introduction to lattice gauge theory and spin systems," Rev. Mod. Phys. **51**, 659 (1979).
[15] M. Serbyn, Z. Papić, and D. A. Abanin, "Quantum quenches in the many-body localized phase," Phys. Rev. B **90**, 174302 (2014).
